# Remarks on the Effects of Antimonial Powder, in Cases of Effusion into the Head

**Published:** 1807-06

**Authors:** William Stoker


### Remarks on the Effects of Antimonial Powder, in Cases of Effusion into the Head; by William Stoker, M. D.

NOTWITHSTANDING the acknowledged assistance which science imparts to art, it must be admitted, that some of the most valued improvements have been made by persons unacquainted with the principles of the subject of their discoveries; on this account, with the varied and extensive range of the medical art, and the bold and frequent experiments which are made in it, popular reports of improvements in the application of means to the cure of diseases, deserve some attention.

Among the useful discoveries which cannot be claimed by the scientific Professors of Medicine (I believe,) may be be ranked antimonial or James's powder as a remedy for hydrocephalus internus, it having been first boldly tried in that disease, and recommended by the Rev. Singleton Harpur.

The profession, character, and education of this gentleman, as well as the frequent opportunities he must have had, when visiting persons in the worst states of sickness, to apply a last resource, claimed in my opinion a trial of a medicine, of which (I understood) he had reported so favourably, *as a Remedy* for a disease, which had been hitherto almost uniformly an opprobrium medicinæ; and as these trials appeared often favourable, I will faithfully, and briefly as I can, state some observations on them, with the cases annexed in which they were made. I have been induced to do so, from not knowing of any medical writer on hydrocephalus, who mentions this remedy with any degree of confidence.

Though the modus operandi of medicines must be confessed to be very imperfectly understood; yet the known effect of antimonial powder when taken internally, appears to me to afford a just rationale of the action of this Remedy, in cases of effusion into the head; for the first sensible effect of this powder on the human system is nausea, proportioned to its dose, during the continuance of which the circulation is slower, the determination of blood to the head is much lessened, as is marked by the paleness of the face, a general and equal softness of the surface succeeds, the extreme vessels both exhalent and absorbent seem restored to a healthy state, and an equable evacuation by excretory and secretory vessels follows. Such effects must considerably counteract any disposition to partial accumulation, and admit of the increased action of the absorbents of the brain, when restored to their healthy capability, and stimulated by the unusual pressure of the exhaled fluid.

How far this rationale will account for the changes in the disease which occurred under the use of this medicine, is freely left for the consideration of the reader, who may be assured, that reports of the cases which led to favourable conclusions respecting this remedy shall not be strained to meet the theory. It is, however, necessary, I think, to premise answers to some objections, which may be made to those cases, as affording any evidence of the good effects of antimonial powder in hydrocephalus internus. 1st. The use of other means at the same time, to which the relief experienced might be attributed. To this

I answer

I answer, that I did not feel justified to make a mere experiment on a human subject, but that I had seen all the other remedies tried in similar cases without any good effect; calomel, indeed, seemed to assist its operation in some instances, where the state of the bowels admitted of that addition, but did not appear to me to be absolutely necessary. 2dly. It may also be objected, that as most of these were cases of effusion into the brain consequent to typhus fever, they do not prove antimonial powder to be even sometimes a cure for idiopathic hydrocephalus. But it will appear, that all the symptoms which mark that diseased state, except the regularity of its stages, were present in these instances, and hence, that the accounts of complete cures even of hydrocephalus by this remedy, are rendered at least probable.

The cases of effusion into the head, in which evident relief first appeared to me to follow the use of antimonial powder, were those of well-marked hydrocephalus internus, which occurred in 1799 and 1800. As this remedy was tried after all the other means that I had known were used without any good effect, the hope of the recovery of the patients was so faint that I did not take notes; and after such a lapse of time cannot attempt to state minutiæ; but the relief, which was very evident, is strongly impressed on my memory. In one of these instances, that of a child about a year old, the symptoms were so far mitigated, that a confident expectation of complete recovery was entertained for several days; it however relapsed, and died. On dissection it appeared to Dr. Wright, who was present, and to myself, that the ventricles of the brain were enlarged to three times their natural capacity, and yet they did not contain more than a tea spoonful of fluid. From this appearance I supposed that the favourable change which followed the use of the antimonial powder, was owing to the absorption of the effused fluid, and that death was caused by the debility incident to so long an illness, and also by the injury done to the substance of the brain.

*The following Cases are extracted from my Journal in the Fever Hospital and House of Recovery, Cork Street.*

Mary Osborne, æt. 18, was admitted August 26, 1805.—She had been ten days previously ill. The commencement of her illness was attended with the usual symptoms which denote the first attack of synochus. On admission into the hospital, she complained chiefly of restlessness, pain of the head and limbs; the skin was hot and dry, tongue brown, only one scanty stool in sixteen hours, the belly not fuller than natural, but sore; pulse 120. Three grains of calomel were ordered, and if no operation followed in three hours, a clyster to be thrown up, which was to be repeated in the evening; to take also a saline effervescent mixture every third hour.

27th. No rest, frequent returns of delirium, the cheeks occupied by a circumscribed flushing, eyes protruded and heavy, skin hot and dry, tongue brown, thirst not urgent, complains much of pain and tightness of head; four copious dark stools, belly soft, urine turbid, pulse 128, and small; some cough. Ordered to repeat the clysters and saline draught, to have barley water for drink, infusion of linseed for the cough, and in the evening, should delirium increase, the head to be shaved and washed with tepid water and vinegar. Wine, five ounces.

28th. Slept two hours after the head was shaved and washed, and has been less delirious; face less flushed, belly softer, little thirst, is at present however rather comatose; eyes heavy, the pupils contracting very tardily on the application of strong light; skin cooler, tongue parched; four dark stools, urine natural; pulse 112.

The washing of the head, barley water, and infusion of linseed were ordered to be continued.

Antimonial powder, two grains, in a pill, to be given immediately, and repeated in the evening: a clyster in three hours, and again in the evening; and if delirium should be violent, a blister to be applied to the nape of the neck. Wine, five ounces.

29th. Some rest, and she complains of the pain of the blister; skin cool, tongue cleaning at the edge; two copious dark stools, belly full, urine natural, pulse 100.— To take immediately two grains of calomel, and in three hours the following draught: Infusion of senna 1 ounce, tincture of senna half an ounce, Rochelle salt 3 drachms, penny royal water 1 ounce. To be repeated in the evening if necessary. Wine, five ounces.

30th. Very comatose thro' the night; urine and stools passed unconsciously, tongue brown, and with a smooth surface; appearance of eyes very little changed. Calomel and purging draught to be repeated, and a blister applied in the evening to the occiput. Wine, eight ounces. To take the antimonial powder four times daily.

31st. Comatose through the night, though the blister rose well; feels it however this morning; seems rather more more lively, and the eyes more sensible to the impression of light; one stool, belly soft, urine copious, skin cool, tongue soft, pulse 90. The antimonial powder to be repeated, and if the coma continues, sinapisms to be applied to the legs in the evening.

Sept. 1st. The sinapisms were applied; was very restless; complains chiefly of a feeling of cold, apparently affecting her feet; they are however, of natural heat; her eyes and countenance have acquired their natural appearance; takes and swallows her medicine better than before, rises to make water and to stool; two stools of a scybalous appearance; pulse 96. Antimonial powder to be repeated. Wine, five ounces.

Sept. 2. Some rest; complains to-day of tinnitus aurium, and also of a more general sensation of noise in the head; countenance has assumed the natural appearance; belly free, pulse 72. The remedies to be repeated. Wine five ounces.

Sept. 3d. Convalescent. Remedies to be repeated.

Sept. 4th. Her recovery was regularly progressive until the 8th, when she was free from complaint, requiring nothing more than the bowels to be kept free by gentle laxatives.

Thomas Casey, a labourer, aged thirty-three years, was admitted into the Fever Hospital in Cork-street, on the 24th of December, 1806; had been three weeks ill.—Report on admission: Delirious; can give no account of previous symptoms, and seems generally agitated on attempting to give a coherent answer to the questions asked him; complains much of his head, to which he frequently applies his hands; tosses his arms much, eyes suffused, skin hot with numerous petechiæ, tongue soft, but brown; no stool since last night; belly soft and not full, breathing hurried, pulse 124, and strong. The head to be shaved, and washed with camphorated spirits of wine; a bolus to be administered of rhubarb grs. xiii. calomel grs. ii. muc. g. arab. q. s. and if necessary, after three hours, a common clyster. When the body is opened, antimonial powder grs. ii. every third hour, and to drink at pleasure, infusion of linseed.

25th. Some rest in the night; five copious brown stools; other symptoms little changed. All the remedies to be repeated, and a blister applied to the nape of the neck.

26th. Little rest, face flushed, pupils dilated, eyes staring; two stools, p. 100. These symptoms nearly as noted on the 24th inst. A blister to be applied to each side of the head, and to take immediately the following draught: Castor oil six drachms, tincture of senna half an ounce, penny royal water two ounces.

27th. Continue. Repeat the antim. powder, and take camphor mixture half an ounce every hour.

28th. No rest, eyes somewhat fixed, pupils much dilated, and do not contract on exposure to a strong light; is affected with nearly complete opisthotonos; had strabismus through the greater part of the night, constant tremors and frequent muscæ volitantes, urine copious, two thin stools, belly soft and empty, p. 112, and feeble. To take immediately, tincture of opium gtts. iv. in half an ounce of camphor mixture, and to repeat to the third time. If the tremors continue, let the back be rubbed with camphorated oil; to have a bolus of calomel ii grs. antim. powder iv grs. and let the antim. powder, in the quantity of iv grs. be repeated every third hour.

29th. Little change till within the last hour, when he expressed relief, and fell into a sleep, which continues; he lies without any apparent uneasiness. Every thing to be repeated as yesterday. Wine, five ounces.

30th. Slept quietly through the greater part of yesterday and last night, countenance more natural, less opisthotonos and strabismus, pupils still dilated, little tremor, belly free, urine copious, still passed unconsciously, pulse 100. Antim. powder to be repeated. Wine, five ounces.

31st. Quiet sleep through the night, and awoke much relieved, speaks and answers cheerfully; no strabismus; pupils of their natural size, and contract readily on the approach of light; no uneasiness at head, can bend forward with ease; drinks well; one copious dark stool, of which he was conscious, and also of making urine, which is plentiful but turbid, and deposits much sediment. The remedies to be repeated.

Jan. 1st. Better in every respect; no return of complaint of the head; skin warmer than natural but soft, pulse natural; one thin yellow stool, belly soft and empty. The remedies to be repeated. Wine, five ounces.

2d. Convalescent. Remedies to be repeated.

3d. Remedies omitted. His recovery, though slow, was complete.

Andrew Magill, aged 40, a taylor, was attacked on August 24, 1805, with the symptoms of fever, particularly with head-ach. He was admitted into the hospital on the the 30th. He then complained principally of pain of his head and want of sleep; his pulse was 120 and small, skin moist, thirst great, tongue covered with a yellowish fur; he had no appetite, and the bowels were costive. He was ordered to take three grains of antimonial powder, and an injection was directed.

31st. Had slept but little; some perspiration had taken place, particularly about the head; he had also some hiccup; complains principally of weakness; is affected with a cough, attended by a considerable expectoration mixed with blood; pulse 126 and small, skin hot, bowels costive. Six ounces of wine, a mucilaginous infusion to be taken occasionally, and an injection, were directed.

Sept. 1. Symptoms somewhat mitigated, cough and expectoration inconsiderable; since last visit had experienced some degree of tinnitus aurium; pulse still frequent, and small; other functions as before, but it was necessary to open his bowels by an injection. The symptoms appeared to decline until the 4th, on which day he made no complaint; his countenance was lively, pulse reduced in frequency to 106, but the skin was still hotter than natural, tongue brown and dry, and bowels requiring the use of gentle laxatives and injections. On the 5th he appeared in a state of stupor, and could not answer when spoken to; his eyes were fixed, and desponding, the left being much suffused; pulse 112, and full, skin hot, tongue dry and clean at the edges; his belly felt somewhat distended; he had one alvine evacuation; his head was directed to be blistered, and he began the use of half an ounce of yeast every three hours.

6th. Could not answer when spoken to; subsultus tendinum and appearance of eyes as yesterday; was unable to protrude his tongue; bowels costive. The wine was continued, blisters applied to the forehead and behind the ears, and he was ordered a pill of calomel and antimonial powder every five hours.

7th. Some difficulty of swallowing occurred during the night; when spoken to, he made some exertion. but was unable to answer; appeared sensible to the attempts made to rouse him from the state of stupor; his eyes were watery, and the pupils of them much dilated; pulse 105, and firm; had passed one large evacuation after the second pill; urine voided in bed.

Under these circumstances the tendency to effusion into the head being evident, he was ordered three calomel and antimonial pills, which were to be taken every four hours, and his head was again directed to be blistered.

8th. He had remained in the comatose state since the last visit till morning, when he complained much of pain of his head; he answered readily when spoken to, and appeared quite collected; considerable subsultus tendinum prevailed; eyes watery, but the pupils were less dilated; pulse 116, skin cool, tongue becoming clean at the edges, and he could protrude it; his bowels had been once opened. The wine was continued, and one antimonial and calomel pill given.

9th. Made no complaint; eyes lively and natural; a gangrenous spot appeared on the os sacrum; pulse 114, small, skin cool, tongue becoming white; had passed eight or nine large stools; urine copious. The chalk mixture was directed with a small quantity of tincture of catechu and opium.

From this time the symptoms continue to decline. On the 12th he complained of soreness of his gums, which continued for some days; the gangrenous spot gradually healed, and he completely recovered. He was dismissed on the 4th of September, free from complaints.

